# Separation of function mutants underline multiple roles of the Srs2 helicase/translocase in break-induced replication in *Saccharomyces cerevisiae*

**DOI:** 10.17912/micropub.biology.001369

**Published:** 2024-11-07

**Authors:** Matteo Di Terlizzi, Giordano Liberi, Achille Pellicioli

**Affiliations:** 1 Dipartimento di Bioscienze, University of Milan, Milan, Lombardy, Italy; 2 Istituto di Genetica Molecolare "Luigi Luca Cavalli Sforza", National Research Council, Pavia, Italy

## Abstract

All cells are commonly exposed to DNA double-strand breaks (DSBs), which must be properly repaired to avoid genomic instability. Break-Induced Replication (BIR) is a Homologous Recombination subpathway, which repairs DSBs resulting in mutagenesis, chromosome translocations and loss of heterozygosity. In budding yeast, the Srs2 DNA helicase/translocase plays both anti- and pro-recombination roles. Interestingly, Srs2 activities are required to support BIR completion. Here, we employ a interchromosomal BIR assay in *S. cerevisiae* to characterize Cdk1-dependent phosphorylation, ATPase and helicase activities of Srs2. Our results further expand our understanding of the multifaced role played by Srs2 in DSB recombination repair.

**Figure 1. Characterization of srs2 mutants in BIR repair:**
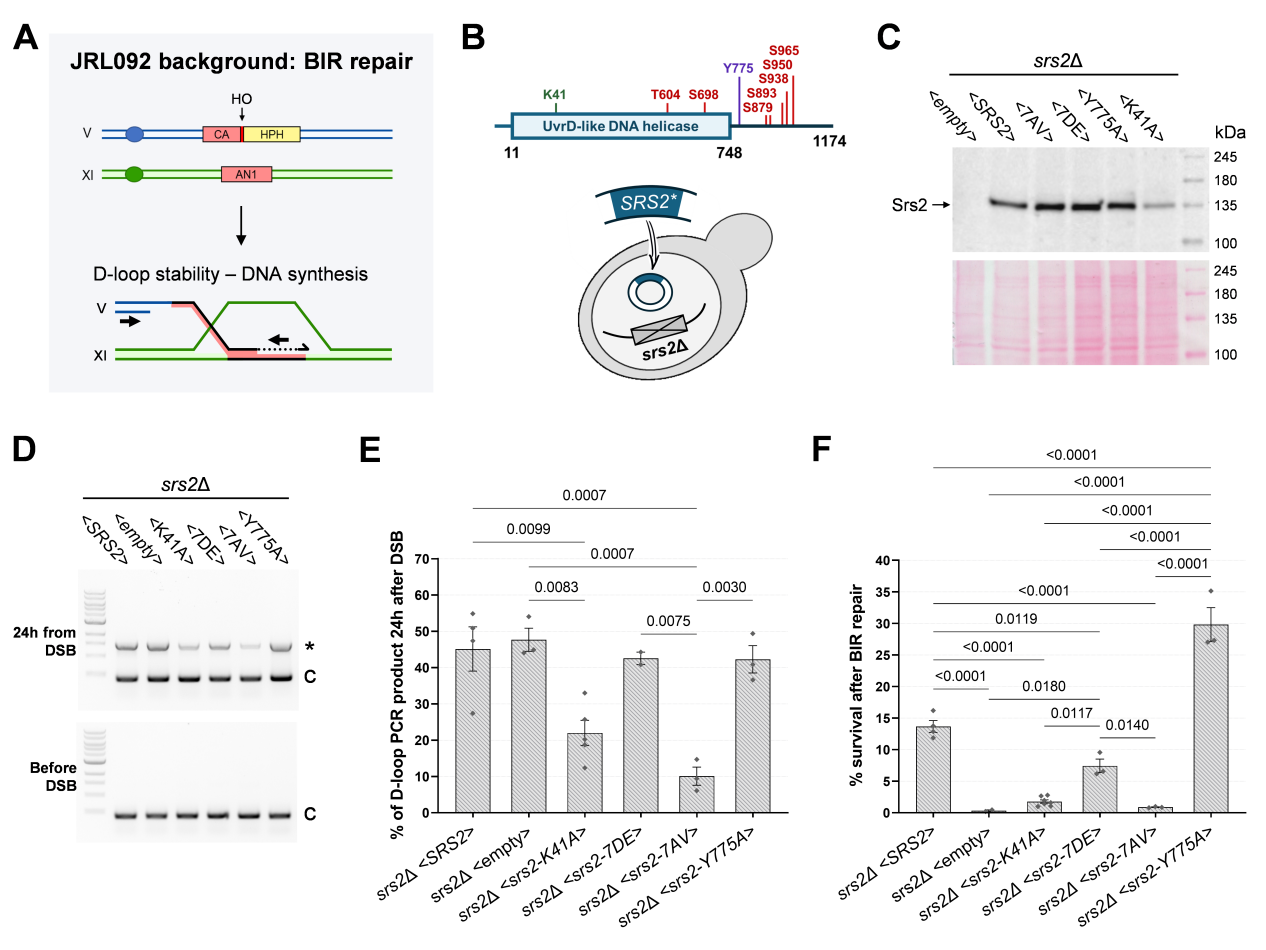
**(A)** Scheme representing JRL092 background (Lydeard et al, 2007). A single DSB is induced in Chr. V, downstream of the truncated *CAN1 ^1-1446^* gene. Recombination occurs with a truncated *CAN ^289-1773^* fragment integrated into Chr XI, 27 kb from the telomere. A PCR-based assay can discriminate between D-loop extension (PCR signal) and failed repair initiation (no PCR signal), using the primers represented as black arrows. **(B)** Scheme of the *SRS2* expression system. Yeast cells are deleted for the endogenous *SRS2* gene and transformed with a centromeric plasmid carrying the sequences of wild-type or mutated *SRS2* alleles, expressed under their endogenous promoter. A schematic representation of Srs2 shows the mutated residues and their position: K41 (green) and Y775 (purple) are located respectively inside the core helicase domain and immediately downstream of it. The seven Cdk1 target Serine/Threonine (red) are mostly located in the unfolded C-terminus and mutated to Alanine/Valine or Aspartate/Glutamate in *srs2* -7AV or *srs2* -7DE alleles, respectively. **(C)** Srs2 levels were assessed by Western Blotting (top) with α-Srs2 antibodies. Cells were grown O/N in selective SC-TRP + Raff medium and collected in exponential phase. Ponceau-S staining of the nitrocellulose membrane (bottom) was acquired to evaluate sample loading. **(D)** Example of gel electrophoresis of PCR products from the D-loop extension assay. **c** : control amplification at uncut *TLC1* locus. * : D-loop extension amplification. **(E)** Quantification of the D-loop extension PCR signals normalized on the correspondent control *TLC1* PCR signals for each strain. Data represent mean ±SEM of at least two independent biological replicates. **(F)** Viability of JRL092-derived cells under continuous HO expression. Data represent mean ±SEM of at least two independent biological replicates.

## Description

Double-strand breaks (DSBs) can be repaired by error-free Homologous Recombination (HR) or error-prone end-joining mechanisms. HR requires the resection of both DSB ends leading to the formation of 3' end single strand (ss) DNA. Upon the binding of multiple factors, including the ssDNA binding protein Rad51, the nucleofilament invades a donor homologous DNA sequence. A transient DNA structure called D-loop is then formed to trigger DNA synthesis repair by exploiting the invaded donor as template (Kowalczykowski, 2015) .

A subpathway of HR takes place when only one of the two DSB ends invades the donor and the newly DNA synthesis is extended till the telomere. Since DNA synthesis is sustained by a migrating replication bubble, this subpathway is called Break-Induced Replication (BIR) (Kramara et al, 2018) . BIR generates a landscape of genomic rearrangements and mutation clusters often reported in cancer cells and is employed by ~15% of tumours to elongate shortened telomeres during ALT pathway (Hoang and O’Sullivan, 2020). Yeast Srs2, a member of the Sf1a helicase family, disassembles Rad51 nucleofilament and limits HR acting as a translocase (Krejci et al, 2003; Veaute et al, 2003) . However, in the absence of Srs2, certain HR subpathways are impaired (Meir and Greene, 2021; Niu and Klein, 2017) , including BIR (Elango et al, 2017) . This suggests that Srs2 plays also a positive role in HR.

Importantly, several *SRS2* mutants have been characterized for their impact on HR. The ATPase defective allele *srs2* -K41A, which is impaired in both translocase and helicase activities (Krejci et al, 2004) , similarly to *srs2* Δ improves D-loop stability (Piazza et al, 2019) , and causes the formation of toxic JM (Elango et al, 2017) . The *srs2* -Y775A allele was recently discovered as a separation-of-function mutant, which retains translocation activity for Rad51 stripping, but is defective in DNA duplex unwinding (Meir et al, 2023) . Importantly, *srs2* -Y775A displays unaltered HR efficiency and outcomes, suggesting that Srs2 helicase activity is dispensable for recombination *in vivo* . Srs2 is phosphorylated in Cdk1-dependent manner in response to DNA damage (Liberi et al, 2000) . The *srs2* -7AV allele, which is mutated in the 7 Cdk1 consensus sites (T604V, S698A, S879A, S938A, S893A, S950A, S965A) (Chiolo et al, 2005) , is proficient in Rad51 nucleofilament disassembly, while it is unable to complete a step in HR after strand invasion. Conversely, the phospho-mimetic *srs2* -7DE does not show any defects in HR (Saponaro et al, 2010) .

Here, we employed the JRL092 background (Lydeard et al, 2007) to test the BIR phenotypes in the *SRS2* mutants described above. In this system, a single DSB is induced on Chr. V by overexpressing HO endonuclease and the break can be repaired mainly by BIR using a 1157 bp homology inserted on Chr. XI, located at 27 kb from the telomere ( Figure 1A ). Failure in completing BIR results in cell lethality (Lydeard et al, 2007) . A centromeric plasmid carrying the full length *SRS2* or the mutants described above was introduced in *srs2* Δ JRL092 strain. An empty vector was used as a control ( Figure 1B ). Firstly, we tested the level of Srs2 variants in exponentially growing cells by Western Blotting. The level of Srs2 protein variants is comparable to wild-type, except for Srs2-K41A, which was reduced as previously reported (Keyamura et al, 2016) ( Figure 1C ). We then evaluated the initiation of DNA synthesis from the D-loop through a previously described PCR technique ( Figure 1A ). In particular, the amplification signal is observed if 292 bp are synthesized from the invading strand of a stable D-loop. While *SRS2* deletion or helicase-defective *srs2* -Y775A allele did not impair D-loop extension at 24 hours from DSB induction, interestingly the *srs2* -K41A mutant displayed a ~2-fold reduction in PCR signal compared to *SRS2* ( Figure 1D, E ). Phospho-null *srs2* -7AV was also extremely defective in D-loop extension (~4.5-fold decrease), while the correspondent phospho-mimetic mutant *srs2* -7DE was comparable to wild-type ( Figure 1D, E ). These results indicate that the helicase, but likely not the translocase activity of Srs2, is dispensable for D-loop-mediated initiation of DNA synthesis repair. Moreover, our results also imply that Cdk1-dependent phosphorylation does not modulate Srs2 helicase activity in BIR. One hypothesis is that *srs2* -K41A and *srs2* -7AV defects shown here are caused by Srs2 protein persisting at the D-loop, which then cannot be properly extended. Consistently, during ectopic gene conversion assay Srs2-7AV was reported to accumulate at donor, even 24 hours after DSB (Saponaro et al, 2010) . Similarly to Srs2-7AV, also the translocase defective Srs2-K41A protein could not be timely dislodged from D-loop, thus impairing the extension. While the Srs2-K41A protein likely remains stacked on DNA because unable to translocate, the Srs2-7AV could persist on DNA intermediate by uncharacterized mechanism.

Finally, the capability to carry out the full BIR process was assayed by cell survival upon DSB induction. As expected, both *srs2-* K41A and *srs2-* 7AV mutants, which were inefficient to carry out D-loop extension, displayed high cell lethality upon DSB induction ( Figure 1F ). Although D-loop can be extended in the absence of Srs2 ( Figure 1D, E ), *srs2* Δ mutant cells cannot survive ( Figure 1F ), which can be explained by toxic JM accumulation during repair synthesis as it was reported in a different BIR assay (Elango et al, 2017) . The *srs2-7DE* mutant also showed reduced viability compared to wild-type (1.84-fold), despite it is proficient in the D-loop extension. These results suggest that Cdk1-mediated phosphorylation of Srs2 may control different steps of BIR, and its deregulation prevents proper synthesis start ( *srs2* -7AV) or a later step, such as JM resolution ( *srs2* -7DE). Another scenario can be observed in *srs2* -Y775A mutant, which displayed higher viability (~2.2-fold) upon DSB induction compared to wild-type cells ( Figure 1F ). These results suggest that Srs2 helicase activity is not required to complete BIR, including the JM resolution step, but on the contrary, it may restrain the process through an uncharacterized mechanism.

This study expands our knowledge on the role played by Srs2 in BIR, and it may be of relevance to understand the deregulation of ortholog DNA helicase/translocase in cancer and to suggest therapeutic targets.

## Methods

**Strain preparation and growth conditions:** All strains are derivatives of JRL092 background (Lydeard et al, 2007) . Deletion of *SRS2* was obtained by one-step PCR protocol (Longtine et al, 1998) and confirmed by PCR. Ycplac22 centromeric plasmids expressing *SRS2* , *srs2* -K41A, *srs2* -7AV and *srs2* -7DE alleles have been previously described (Saponaro et al, 2010) . Ycplac22 *<srs2-Y775A* > was obtained by site-directed mutagenesis (Diani et al, 2009) from Ycplac22 *<SRS2>* and checked by sequencing. To avoid plasmid loss, cells were always grown at 28°C in SC-TRP medium enriched with either Glucose 2% (Glu), Galactose 2% (Gal) or Raffinose 3% (Raff).

**D-loop extension assay:** This protocol was performed as previously described (Ferrari et al, 2020; Lydeard et al, 2007) . In brief, cells were grown O/N in SC-TRP + Raff medium till reaching early exponential phase. Cells were normalized to 10 ^7^ cells/mL and Galactose 2% was added to the growing medium to induce HO expression. Cells were collected before Gal addition and after 24 hours. A home-made protocol adapted from (Boeke et al, 1985) was employed for genomic DNA extraction. Purified DNA is quantified by EtBr staining after gel electrophoresis and 25 ng were used as template for PCR amplification within the linear range (28 cycles). A schematic representation of the PCR assay with the primers employed is represented in Figure 1A . PCR amplification of a control uncut locus *TLC1* on Chr. II was performed in the same reaction mix as a reference. PCR products were subjected to electrophoresis in 0.8% agarose gel and EtBr signal was acquired. The percentage of D-loop extension was calculated as the ratio between signal of the D-loop product and signal for *TLC1* .

**Western Blotting:** Proteins are extracted as previously described (Foiani et al, 1995) and separated by electrophoresis on 10% Acrylamide gel. Proteins are transferred on nitrocellulose membrane and loading was evaluated with Ponceau-S staining. Western Blotting was performed using polyclonal α-Srs2 goat antibodies (sc-11991, SantaCruz).

**Viability assay:** JRL092-derived strains were grown O/N at 28°C in SC-TRP + Raff till early exponential phase. Cells were diluted and suitable amounts were spread on SC-TRP + Glu and SC-TRP + Gal plates. Plates were incubated 4 days at 28°C before counting growing colonies. Viability was calculated as the ratio between the number of colonies on Gal-containing medium and the total amount of cells plated, obtained from the number of colonies growing on Glu-containing medium.

**Statistical analysis:** Statistical analysis was performed using GraphPad Prism 9 software. *P* -values were calculated by unpaired parametric statistics through one-way ANOVA, corrected for multiple comparisons through Šidák method.

## Reagents

**Yeast strains:**

**Table:** 

**Strain name**	**Original strain**	**Genotype**	**Reference**
Y4797	JRL092	*hoΔ mat::hisG hmlΔ::hisG HMRa-stk ura3Δ851 trp1Δ63 leu2Δ::KAN ade3::GAL10::HO; can1, 1-1446::HOcs::HPH* on chr V; *ykl215c::LEU2::can1Δ289* on chr XI	(Lydeard et al, 2007)
Y4990	Y4797	*srs2* ΔNAT	This study
Y5049, Y5050	Y4990	*<empty, TRP1, CEN* >	This study
Y5019, Y5020	Y4990	*<SRS2, TRP1, CEN* >	This study
Y5014, Y5039	Y4990	*<srs2-K41A, TRP1, CEN* >	This study
Y5015, Y5016	Y4990	*<srs2-7AV, TRP1, CEN* >	This study
Y5017, Y5018	Y4990	*<srs2-7DE, TRP1, CEN* >	This study
Y5262, Y5263	Y4990	*<srs2-Y775A, TRP1, CEN* >	This study

**Plasmids:**

**Table:** 

**Plasmid Name**	**Original plasmid**	**Genotype**	**Reference**
B486	CB1015	Ycplac22 ( *CEN; TRP; srs2-K41A* )	(Chiolo et al, 2005)
B487	CB1016	Ycplac22 ( *CEN; TRP; srs2-7AV* )	(Chiolo et al, 2005)
B488	CB1070	Ycplac22 ( *CEN; TRP; srs2-7DE* )	(Saponaro et al, 2010)
B489	CB1071	Ycplac22 ( *CEN; TRP; SRS2* )	(Chiolo et al, 2005)
B491	Ycplac22	Ycplac22 ( *CEN; TRP; empty* )	(Chiolo et al, 2005)
B517	B489	Ycplac22 ( *CEN; TRP; srs2-Y775A* )	This study

**Oligonucleotides:**

**Table:** 

**Name**	**Sequence**	**Technique**	**Position**	**Reference**
P1	GAGGATACGTTCTCTATGGAG	D-loop extension assay	*CAN1* on Chr V	(Ferrari et al, 2020)
P2	GTCTTTGGTTCATGATCTTCCC	D-loop extension assay	*CAN1* on Chr XI	(Ferrari et al, 2020)
Srs2-Y775A TOP	CGAATAGTATCAAAAAACTAGCCCGAATTTTGAACAAAAAGCCG	Site-directed mutagenesis	*SRS2* gene	This study
Srs2-Y775A BOT	CGGCTTTTTGTTCAAAATTCGGGCTAGTTTTTTGATACTATTCG	Site-directed mutagenesis	*SRS2* gene	This study

**Antibodies:**

**Table:** 

**Name**	**Type**	**Species**	**Brand**
α-Srs2 (yC-18, sc-11991)	Polyclonal	Goat	Santa Cruz Biotechnology
α-Goat (Secondary, HRP-conjugated)	Polyclonal	Rabbit	Invitrogen
